# A Meta-Analysis on the Relationship between Exposure to ELF-EMFs and the Risk of Female Breast Cancer

**DOI:** 10.1371/journal.pone.0069272

**Published:** 2013-07-15

**Authors:** Qingsong Chen, Li Lang, Wenzhe Wu, Guoyong Xu, Xiao Zhang, Tao Li, Hanlin Huang

**Affiliations:** 1 Guangdong Prevention and Treatment Center for Occupational Diseases, Guangzhou, China; 2 National Institute of Occupational Health and Poison Control of the Chinese Center for Disease Control and Prevention, Beijing, China; Health Canada and University of Ottawa, Canada

## Abstract

**Objective:**

To comprehensively analyze the relationship between exposure to extremely low frequency electromagnetic fields (ELF-EMFs) and the development of female breast cancer.

**Methods:**

Reports of case-control studies published from 1990 to 2010 were analyzed. The quality effect model was chosen to calculate total odds ratio (*OR*) depending on the data in studies and quality scores. Subgroup analyses were also performed by the situation of menopause, estrogenic receptor and exposure assessment respectively.

**Results:**

For all 23 studies the *OR* was 1.07, 95% *CI* = 1.02–1.13, for estrogen receptor positive subgroup,*OR* = 1.11, 95% CI = 1.03–1.20; for premenopausal subgroup, *OR* = 1.11, 95% CI = 1.00–1.23. The results of other subgroups showed no significant association between ELF-EMF and female breast cancer.

**Conclusion:**

ELF-EMFs might be related to an increased risk for female breast cancer, especially for premenopausal and ER+ females. However, it's necessary to undertake better epidemiologic researches to verify the association between ELF-EMF and female breast cancer due to the limits of current study, especially the one on exposure assessment.

## Introduction

Extremely low frequency electromagnetic fields (ELF-EMFs) are 0–300 Hz electromagnetic fields that are mainly generated by power transmission lines, power equipment and appliances. The possible association between ELF-EMFs and different cancers such as brain tumors, leukemia, and breast cancer [1]–[3] has been discussed widely since 1979 when an epidemiologic study by Wertheimer and Leeper suggested a possible link between ELF-EMFs and childhood leukemia [4]. Breast cancer is the most prevalent malignant disease in women, and its incidence continues to increase. In 1987, Stevens suggested that ELF-EMFs and visible light at night (around 1015 Hz) may increase the long term risk of breast cancer[5]. To date, numerous scholars around the world have examined the correlation between ELF-EMFs and the development of breast cancer.

The methods on choosing the objects of a study were similar in terms of previous case-control studies on ELF-EMFs and breast cancer. The cases were often chosen from the registration systems related to cancer or hospital case reports. There were various ways of choosing objects to form control groups, the controls of some studies were chosen from other cancer patients and some were chosen from a group of people who live in the same area by random sampling. Meanwhile age and gender were often taken into consideration to form a control group. The studies on the association between ELF-EMF and development of female breast cancer have not reached a consensus yet. The accuracy of study on the correlation between ELF-EMF and development of female breast cancer was mainly attributed to the assessment of exposure and the identification between exposure group and un-exposure group. The electromagnetic fields were everywhere, therefore it's inevitable that everyone was exposed to electromagnetic fields to a certain degree. Although some studies have taken life and occupations into consideration, most of the studies only focused on one aspect of life habits, resident environment or work environment in terms of exposure assessment due to its complexity. The exposure groups were often confirmed in the following ways: 1.using electric heating equipment such as electronic blanket or not; 2. exposure level of living environment; 3. work duty or measuring and assessment of working environment; 4. distance from the high voltage power lines; 5. combining some above factors.

Currently there were two teams who had conducted the meta-analysis in regard to the correlation between ELF-EMF and development of female breast cancer. At first, T.C. Erren meta-analysed the case-control studies and cohort studies before 2000 and the results showed the pooled RR from studies in women was 1.12 (95% CI = 1.09–1.15), but variations between the contributing results are not easily attributable to chance (P = 0.0365). Meanwhile T. C. Erren indicated that some research methods might lead to the incorrect assessment of exposure or cases, which has caused the discrepancy of many study results [6]. A total of 15 case-control studies published over the period 2000 to 2009 including 24,338 cases and 60,628 controls were involved in the meta-analysis of Chunhai Chen etc. [7]. The results showed no significant association between ELF-EMF exposure and female breast cancer risk in total analysis (OR = 0.988, 95% CI = 0.898–1.088) and in all the subgroup analyses by exposure modes, menopausal status, and estrogen receptor status.

A large number of scholars around the world have undertaken plenty of studies on the association between ELF-EMF and development of female breast cancer. The method of meta-analysis used in those studies was remarkable. However, Chunhai Chen only summarized the literature from 2000 to 2009 and failed to incorporate many significant studies before 2000. Our study firstly would go through the literature from 1990 to 2012 using the method of meta-analysis. Secondly, because the confounding factors adjusted in the statistical analysis and the methods dividing the exposure groups were different in different literature, our study would undertake statistical analysis through searching the number of exposed and un-exposed individuals in the original cases or controls instead of adopting the adjusted OR or RR value in the literature. The number of samples in different groups was missing in most of cohort studies so that we have just taken all case-control studies into consideration for calculating the total OR value in this study. At last, if using traditional fixed effect model or random effect model, the important role of literature with excellent study design would not be displayed in the Meta analysis study. Considering the diversity of different study design quality, our study would use quality effect model to conduct meta- analysis in which literature with better study design would be prioritized to produce more accurate results.

## Materials and Methods

### 1. Identification of studies and eligibility criteria

Medline, PubMed, EMBASE and Hirewire databases were used to search for studies assessing the relationship between ELF-EMFs and female breast cancer from January 1990 to December 2012 using synonyms and combinations of the terms ‘breast cancer’, ‘breast neoplasm’, and ‘electromagnetic fields’. At first, two reviewers (Q.S. Chen, W.Z. Wu) picked up the case-control studies and cohort studies from the identified articles by reviewing the titles and abstracts. Then they read the full text and chose the studies as objects of study according to the following criteria: (1) the publication was a population epidemiology study on the association between ELF-EMF exposure and breast cancer in females; (2) the papers must offer the size of the samples, number of exposed and non-exposed individuals in cases and controls; and (3) publication language was confined to English. When multiple publications reported on the same or overlapping data, we used the most recent or largest population as recommended by Little et al.

### 2. Data collection and quality assessment

Assessment and division of exposure often was the most important reason leading to difference in the results of studies of correlation between ELF-EMF exposure and breast cancer in females. In the articles covered in this study, the ELF-EMF exposure has been assessed through investigating the usage of electric blanket or position title, divided through measuring and assessing of the electrometric field level in the living and working environment. For the studies assessing exposure level by measuring or calculation, the researchers usually used the group with the lowest exposure as the referent to calculate the OR value and different intensity factors such as 0.1 µT,0.2 µT are used to decide the lowest exposure group in different studies. In this meta-analysis, the group with the lowest exposure was selected as the referent and other groups were put together as exposure group to calculate the total OR value. The all literature mentioned in our study has taken age and region into consideration in choosing cases and controls. The confounding factors adjusted in the statistical analysis (such as age, habit of smoking and drinking, race, menopausal status, and estrogen receptor status) were quite differential in different study. So, the number of exposed and un-exposed individuals in cases and controls were abstracted for meta-analysis instead of copying the adjusted OR or RR value from the literature.

In this study an extracting list on the important information of the literature was made. Two researchers read and analysed the literature individually. They abstracted the key information that includes: the author, the year of publishing, the country of the objects, the number and selecting method of cases and controls, the assessing methods of exposure, matching factors of cases and controls, the year of research, OR value and 95%CI. If the results of the analysis conducted by the two researchers are inconsistent, it will be solved through discussion.

The studies on the correlation between ELF-EMF exposure and breast cancer in females used different research methods, especially on the assessment and dividing of the exposed and un-exposed individual. Considering some studies are more accurate and reliable, the quality assessment method of cases and controls study has been developed based on Newcastle-Ottawa Scale [8]. All studies were assessed from three aspects that included ten indicators in terms of choosing method of cases and controls, comparability of cases and controls and exposure assessment. The Quality scoring criteria was showed in table 1 in which each indicator was given one score and the total score of every study was ten. The quality assessment and scoring of all literature would be conducted by two experts in this field independently. If the results of the analysis are inconsistent, it would be solved through discussion.

**Table 1:** Quality scoring criteria.

Evaluating items	Quality Criteria	Quality Score
**1. Selection**		0–4
1.1 Is the case definition adequate?	Requires some independent validation (e.g. >1 person/record/time/process to extract information, or reference to primary record source such as pathology or medical/hospital records) = 1	
	Record linkage (e.g. International Classification of Diseases ICD codes in database) or self-report with no reference to primary record, or no description = 0	
1.2 Representativeness of the cases	consecutive or obviously representative series of cases = 1All eligible cases with outcome of interest over a defined period of time, all cases in a defined catchment area, all cases in a defined hospital or clinic, group of hospitals, health maintenance organisation, or an appropriate sample of those cases (e.g. random sample)	
	Not satisfying requirements above, or not stated = 0	
1.3 Selection of Controls	community controls (i.e. same community as cases and would be cases if had outcome) = 1	
	hospital controls, within same community as cases (i.e. not another city) but derived from a hospitalized population or no description = 0	
1.4 Definition of Controls	no history of disease (endpoint) = 1If cases are first occurrence of outcome, then it must explicitly state that controls have no history of this outcome. If cases have new (not necessarily first) occurrence of outcome, then controls with previous occurrences of outcome of interest should not be excluded.	
	no description of source = 0	
**2. Comparability**		0–2
1) Comparability of cases and controls on the basis of the design or analysis	study controls for age and region = 1	
	study no controls for age and region = 0	
	study controls for heredity factors = 1	
	study no controls for heredity factors = 0	
**3. Exposure**		0–4
3.1 Ascertainment of exposure	assessment of exposure by measurement or strict caculation = 1	
	assessment of exposure by questionnaire or no description = 0	
	assessment of exposure including environmental, living and occupational exposure = 1	
	assessment of exposure only including one aspect of life or work such as usage of electric heating equipment, work duty and distance from the high voltage power lines = 0	
3.2 Same method of ascertainment for cases and controls	Yes = 1	
	No = 0	
3.3 Non-Response rate	same rate for both groups = 1	
	non respondents described or rate different and no designation = 0	
Total score		0–10

### 3. Statistical analysis

Microsoft Excel was used to organize the initial data and build a database. The quality index of every study was equal to the quality total score of every study divided by ten. Then a quality effect model of MetaXL version 1.3 was applied to analyze the data and calculate the total OR and 95% confidence interval to assessing the relationship between ELF-EMFs and breast cancer in females [9]–[11]. MetaXL implements a process called quality effects model to explicitly address study heterogeneity caused by differences in study quality. This model is a modified version of the fixed-effects inverse variance method that additionally allows giving greater weight to studies of high quality. Considering the difference of exposure assessment method, subgroup meta-analyses by quality effect model were performed according to exposure modes (occupational exposure, residential exposure, blanket exposure and multiple exposures). Considering the effect of hormone to breast cancer, the subgroup analyses also were performed by menopausal status and estrogen receptor (ER) status. Heterogeneity assumption was assessed by Chi-square based Q-test and I-squared test. If P value for Q test <0.10 then refuse Ho, the heterogeneity is significant. An inverted funnel plot was drawn with an abscissa of each study's OR value and an ordinate of the standard error (SE) for publication bias examination.

## Results

Twenty four case-control studies and sixteen cohort studies were collected in this meta-analysis. The number of samples in different groups was missing in most of cohort studies so that we did not take any cohort study into consideration for calculating the total OR value. Two case and control studies were the comments on other studies and one literature incorporated two studies. Eventually we conducted meta-analysis on 22 articles or 23 case-control studies [3], [12]–[32] that could meet the criteria. The characteristics of all eligible studies were showed in the Table 2. There were seven studies[18], [19], [21], [25], [27], [30], [31] that included information about estrogen receptor and nine studies[3], [12], [14], [18], [19], [23], [25], [32] that conducted the investigation on menopausal status in the 23 case-control studies. In our study, there were eight studies[3], [12], [15], [18], [20], [22], [25] that made an exposure assessment through investigating the usage of electric heating apparatus such as electric blanket, seven[11], [13], [21], [25], [26], [29], [30] through working history, five[16], [19], [24], [26], [29] through the exposure level of the living environment, two[21], [30] through measuring and calculating the ELF-EMF levels based on the living and working conditions. In the 23 studies, there were sixteen studies[3], [12]–[15], [17], [18], [20], [22]–[24], [27]–[29], [32] from America, three[19], [21], [31] from Sweden, two[26], [30] from Norway, one from Canada and one from Taiwan. In terms of case selecting, fourteen [3], [15]–[19], [21]–[24], [28], [29], [31], [33] studies selected their cases from cancer registry, other cases were selected from hospitals or other cohort studies. The controls from 19 studies [3], [12], [14], [15], [17]–[19], [21]–[27], [29]–[32] were residents randomly selected from driver's license records, telephone number lists and so on. In the rest four studies [13], [15], [20], [28], The control groups were selected from patients with other cancer or diseases and there was a matching in terms of age and living area in case and control groups.

**Table 2:** Summary of 23 case-control studies on exposure to electric and magnetic fields and breast cancer in females.

No	1st author, year of publication and country	Cases	Controls	Exposure assessment method	The matched factors between cases and controls	Study period	OR (95%CI)	Scores
1	Vena JE(1991), USA	378 postmenopausal women in the Western New York Study of Breast Cancer	438 controls randomly selected from community	Frequency and mode of use of electric blankets	Age, region	1987–1989	0.89(0.67,1.19)	
2	Loomis DP(1994), [^15^] USA	27882 Cases were female residents for 20 years and older at their death in 24 states.	110949 controls were random sample of women who died of any other underlying cause, excluding leukemia and brain cancer.	Job title.	Year of death, age, region	1985–1989	1.36(1.03,1.79)	6
3	Vena JE(1994), USA	290 premenopausal women who were admitted to hospitals in Niagara and Erie counties	289 controls who were residents of the same two counties, randomly selected from the New York State driver's license records	Histories of electric blanket use	Age, region	1986–1991	1.14(0.81,1.59)	5
4	Coogan PF(1996), USA	6888 cases were female residents of four states with incident breast cancer reported to four tumor registries	9529 controls were randomly selected from state driver's license lists and health care telephone number	Job title	Age, region	1988–1991	1.00(0.90,1.11)	7
5	Li C-Y(1997), Taiwan	1980 cases were residents of northern Taiwan reported to the National Cancer Registry of Taiwan	1880 controls were random selection of women with cancers excluded these cancers associated with magnetic field exposure	Measurement and Estimation of magnetic fields in the residencies	Age, sex, and date of diagnosis	1990–1992	1.10(0.93,1.31)	5
6	Coogan FP(1998) [^12^], USA	259 cases were permanent residents of five towns and reported to the Massachusetts Cancer Registry.	738 controls resided in the towns were selected by random digit dialing, lists of Medicare beneficiaries, and death certificates.	Use of electric bed-warming devices and electric heat, Occupational history and residential history	Age, region	1983–1986	0.99(0.74,1.33)	7
7	Gammon (1998), USA	1645 incident cases were residents of one of three US geographic areas with a tumor registry.	1498 controls were identified via random digit dialing	Use of electric blankets	Age, region	1990–1992	1.06(0.92,1.22)	6
8	Feychting M(1998) [^13^], Sweden	669 cases were identified through record linkage to the Swedish Cancer Registry.	669 controls were selected randomly among those who were included in the study base.	The magnetic field at home were assessed through theoretical calculations	Age, lived in the same parish, and lived near the same power line.	1960–1985	1.14(0.86,1.51)	6
9	Zheng (2000), USA	608 Cases either had breast-related surgery at the Yale-New Haven Hospital, or who were residents of Tolland County	609 controls had had breast-related surgery and who were histologically diagnosed with normal tissue or benign breast diseases.	Use of electric blankets	Age, region	1994–1997	0.86(0.69,1.09)	5
10	McElroy JA (2001) [^21^], USA	1,949 cases were identified from state wide tumor registries in Massachusetts, New Hampshire, and Wisconsin	2,498 cases were randomly selected from population lists as controls.	Electric blanket and mattress cover use	Age	June 1994–July 1995	0.97(0.86,1.09)	6
11	Wijngaarden (2001), USA	843 cases were identified through the North Carolina Central Cancer Registry	773 Controls were sampled from lists of the Division of Motor Vehicles and Health Care Financing Administration	Cumulative exposures to magnetic fields were based on a measurement survey.	Age, race	1993–1995	0.94(0.76,1.16)	7
12	Davis S(2002),USA[^23^]	813 cases were identified by the Cancer Surveillance System of the Fred Hutchinson Cancer Research Center.	793 controls were resident and identified by random digit dialing.	Measurements in the home and self-reported measures of at-home electric appliance use.	Race, age and region	November 1992–March 1995	0.99(0.77,1.28)	7
13	Kabat GC (2003a) [^22^],USA	1323 cases were identified from Health Care Financing Administration files was from the Long Island Breast Cancer Study Project (LIBCSP)	1362 controls were from the LIBCSP and controls were residents identified by random digit dialing and Health Care Financing Administration (HCFA) rosters.	Electric blanket use	Age, region	August 1, 1996 to July 31, 1997.	1.11(0.94,1.30)	6
14	Kabat GC(2003b) [^22^],USA	666 cases was from the Electromagnetic Fields and Breast Cancer on Long Island Study (EBCLIS*)	I 557 controls were from EBCLIS	Electric blanket use	Age, region, long-term residents	August 1, 1996 to July 31, 1997.	0.97(0.76,1.23)	5
15	Schoenfeld ER (2003) [^16^], USA	576 cases was from the EBCLIS	585 controls were from EBCLIS	In-home EMF measurements, wire mapping of overhead power lines	Age, region, long-term residents	August 1, 1996, to June 20, 1997.	1.03(0.82,1.30)	7
16	Kliukiene J (2003) [^17^],Norway	99 breast cancer cases from a cohort of Norwegian female radio and telegraph operators.	396 controls from the cohort alive at time of diagnosis.	Calculated based on employment information. Magnetic field measurements	Age, region	January 1961 to the end of May 2002.	1.46(0.78,2.70)	6
17	Labreche F(2003)[^19^], Canada	608 cases were identified from records of pathology departments and cancer registries from hospitals.	667 controls had 32 different types of cancer from the same hospitals.	Job	Age, same hospital	1996–1997	1.22(0.93,1.61)	5
18	London SJ (2003) [^24^], USA	347 cases were identified by linkage to county and state tumor registries in Los Angeles County, California.	286 Controls were selected from a random sample of cohort members without breast cancer at baseline.	Exposure was assessed by means of wiring configuration coding	Race, age and region	1993–1999	1.26(0.87,1.83)	8
19	Zhu K, 2003(2003) [^3^], USA	304 cases lived in one of three Tennessee counties were identified through the Tennessee Cancer Reporting System.	305 controls were selected through random digit dialing.	Electric blanket use	Race, age and county	1995–1998	1.49(0.99,2.23)	6
20	Kliukiene J (2004) [^17^],Norway.	1830 cases of breast cancer were identified in a cohort of women living near a high-voltage power line in Norway.	The 3658 controls were selected randomly from the cohort.	Residential exposure by the lines, occupational exposure by job title, Magnetic field measurements for estimated time weighted average;	Age, region	1986–1996	1.53(1.28,2.85)	9
21	Forssen (2000) UM[^25^], Sweden	440 cases living within 300 meters of transmission lines in the cohort were identified from the Swedish Cancer Registry.	439,One matched control per case at random was selected.	Residential exposure by the power lines, and occupational exposure by job title,	Age, region, type of house, power line	1960–1985	1.02(0.78,1.35)	8
22	Forssen UM(2005), Sweden	18365 cases were identified from the cancer registry gainfully	101973 controls were selected randomly from the study base.	Job-exposure matrix based on personal magnetic field measurements	Rgion	1976–1999	1.04(0.99,1.08)	6
23	McElroy JA (2007) [^20^],USA	6213 cases were identified through the North Carolina Central Cancer Registry.	7390 Controls were sampled from lists of the Division of Motor Vehicles and rosters of Medicare beneficiaries.	Job title	Age, region	1970–2002	1.06(0.99,1.14)	6

*:Women eligible for EBCLIS were those LIBCSP participants who had lived in their current residences for 15 years or more (long-term residents).

**Figure 1:**
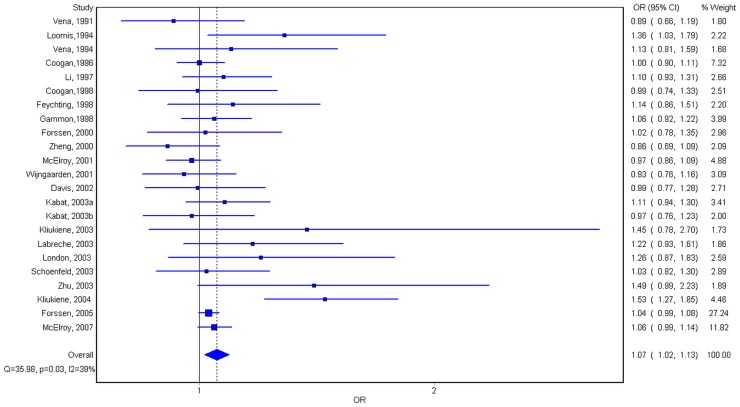
Forest stereogram of the meta-analysis on the association between the exposure to ELF-EMFs and female breast cancer.

From figure 1, the results of sixteen studies, occupied 69.57% of all 23 case control studies, showed OR value was over one and the other OR values of seven studies was under one. Except the results of Kliukiene J and Loomis DP's studies [13], [30] showed a statistic significance for (OR = 1.53, 95% CI = 1.28–2.85), we did not find any statistic difference in other studies. In this study, the results of all 23 case-control studies analyzing by quality effect model showed a significant association between ELF-EMF and female breast cancer, the total OR value and 95% CI were 1.07 and 1.02–1.13. The results of meta analysis of the subgroups showed that for estrogen receptor positive subgroup,OR = 1.11, 95% CI = 1.03–1.20; for estrogen receptor negative subgroup, OR = 0.96, 95% CI = 0.84–1.10; for premenopausal subgroup, OR = 1.11, 95% CI = 1.00–1.23; for postmenopausal subgroup, OR = 1.02, 95% CI = 0.95–1.09; for blanket exposure subgroup, OR = 1.03, 95% CI = 0.95–1.12; for occupational exposure subgroup, OR = 1.08, 95% CI = 1.00–1.15; for residential exposure subgroup, OR = 1.09, 95% CI = 0.97–1.22 and for multiple exposure assessment subgroup, OR = 1.35, 95% CI = 0.97–1.89.

**Figure 2:**
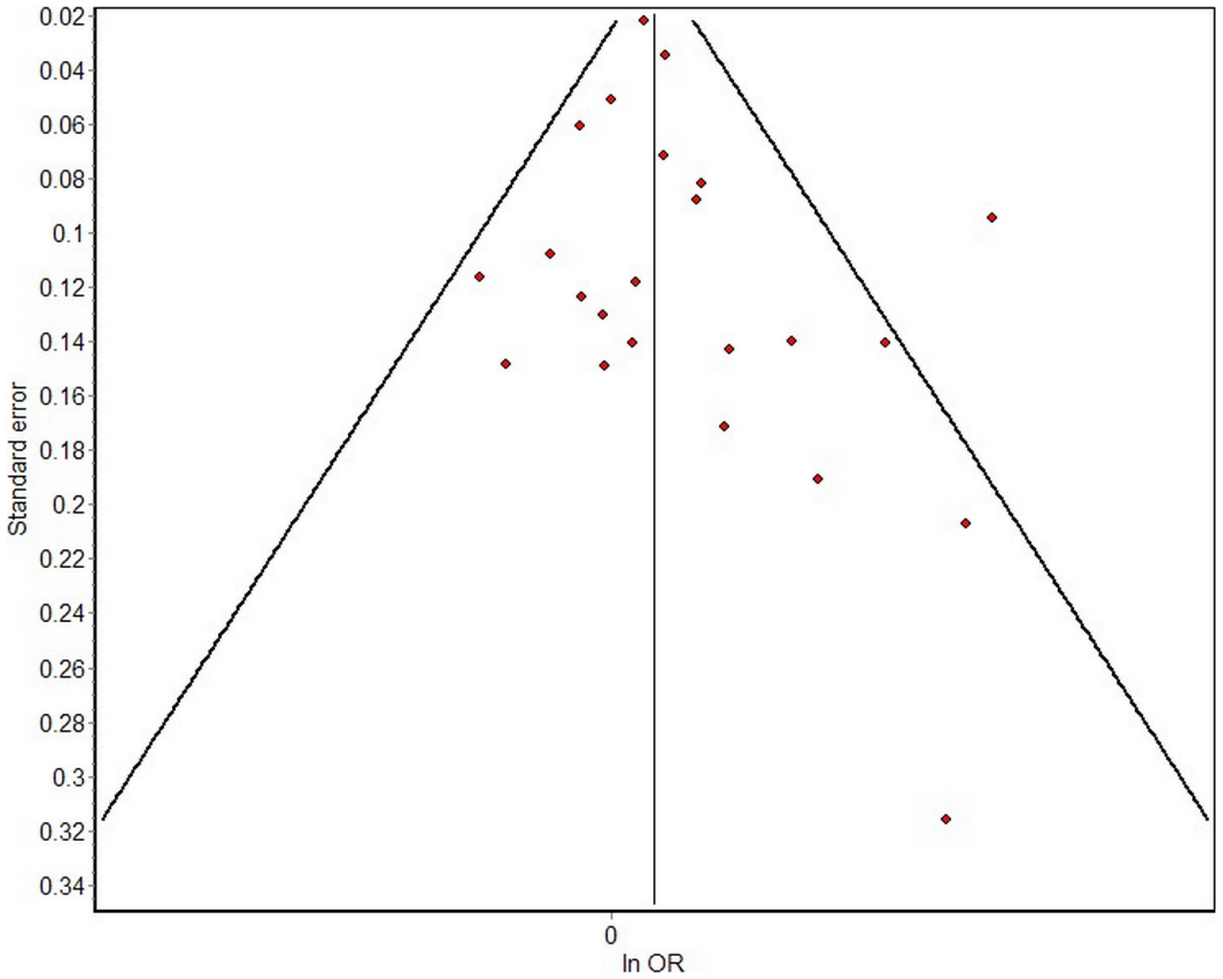
Funnel plot analysis of the selected articles' publication bias.

As shown in table 3, the P value was less than 0.1 and the I2 value was equal to 39% by heterogeneity test for total comparison that indicated the heterogeneity was significant, though the difference was small. For all subgroups, except multiple exposures subgroup in which the heterogeneity was significant (P<0.1, I2 = 82%) the homogeneities of other subgroups were great, P>0.1. MetaXL version 1.3 software was used to draw a funnel plot and to perform a linear regression analysis. Figure 2 presents the data distribution, which was almost bilaterally symmetrical, demonstrating that the bias was small.

**Table 3:** Summary *OR* and 95% *CI* of ELF-EMF exposure and female breast cancer risk.

Group	Study number	*OR*	95% *CI*	*P* **	*I* ^2^
All studies*	23	1.07	1.02–1.13	0.03	39%
Exposure modes					
blanket exposure	8	1.03	0.95–1.12	0.27	21%
occupational exposure*	7	1.08	1.00–1.15	0.24	25%
residential exposure	5	1.09	0.97–1.22	0.83	0%
multiple exposure	2	1.35	0.97–1.89	0.02	82%
ER status					
ER+*	7	1.11	1.03–1.20	0.85	0%
ER–	7	0.96	0.84–1.10	0.54	0%
Menopausal status					
Premenopausal*	9	1.11	1.00–1.23	0.24	22%
Postmenopausal	9	1.02	0.95–1.09	0.60	0%

*P<0.05;

**P value of heterogeneity.

## Discussion

The association between the exposure to ELF-EMFs and the development of female breast cancer has always been a controversial topic in the scientific field. Animal testing has indicated that exposure to ELF-EMFs could increase the risk of cancer. Loscher and others used DMBA to induce breast cancers in mice and used 50 Hz,0.2–1 µT, 10 µT, 50 µT, and 100 µT magnetic fields to irradiate mice 24 hours a day for 13 weeks [33]. A dose-response relationship between cancer rates and magnetic fields was observed in that study. However, Owing to the uncertainties of exposure and confounding factors such as the information on the exposure levels of the case and the control group in most epidemic studies is uncertain, research reports on ELF-EMFs have often demonstrated differences in results. In this studies, two studies showed a relation between exposure to ELF-EMFs and female breast cancer [11], [28], while other 21 studies did not indicate this conclusion[3], [10], [12]–[27], [29], [30]. This meta-analysis comprehensively analyzed twenty three items of case-control studies to examine the relationship between the exposure to ELF-EMFs and breast cancer. As shown in table 3, except multiple exposures subgroup in which the heterogeneity was significant because of the limit of sample size, the homogeneities of other groups were great. The funnel plot also demonstrated that the publication bias of the selected articles was small. The results of this study showed the pooled OR = 1.07,95% CI = 1.02–1.13 by the quality effect model, which indicated that the development of breast cancer might be potentially related to exposure to ELF-EMFs. The result of our study is close to the one of Meta-analysis conducted by T.C. Erren[6] in 2001 in which the pooled RR from studies in women was 1.12 and 95% CI = 1.09–1.15. However, because results from individual studies were very variable and in part contradictory, T.C. Erren concluded the paramount methodological problem inhibiting valid conclusions about an association between EMF and breast cancer was the probable misclassification of exposure and the possible misclassification of the disease itself. Another meta analysis about an association between EMF and breast cancer was published by Chunhai Chen etc. in 2010 and the results showed no significant association between ELF-EMF exposure and female breast cancer risk in total analysis (OR = 0.988, 95% CI = 0.898–1.088). Compared with the above two studies, this study have covered literature from 1990 to 2012 and taken the lowest exposure group (most nearly zero exposure) as the un-exposed groups. This study was also different from the previous studies in which fixed effect model and random effect model were adopted in terms of methods on meta-analysis. This study has used quality effect model to do meta-analysis in which studies with better methods are prioritized. Of course, judging from the quality scoring of the selected 23 studies, exposure assessment was still the most important factor that affected the accuracy and reliability of study.

It is generally accepted that, to date, no research has demonstrated that EMFs directly affect the body to exert a chronic influence on health. The relationships between the EMFs and the environment, body, and other factors have become the major focus for research on health-related issues, especially cancer, caused by EMFs. Many studies have suggested that the occurrence and development of breast cancer were closely related to estrogen, melatonin, and other hormones. As previously discussed, Girgert et al. [34] confirmed the changes in expression of cofactors of the estrogen receptors in human breast cancer cells exposed to low frequency EMFs by in vitro study. This meta-analysis analyzed nine items of premenopausal case-control studies and nine items of postmenopausal studies to examine the relationship between exposure to ELF-EMFs and breast cancer. Through stratification analysis, the OR of the premenopausal subgroup was 1.11, 95% CI = (1.00–1.23) and that of the postmenopausal group was OR = 1.02, 95% CI = (0.95–1.09). The seven studies with estrogen receptor information were analyzed, the ER+ (estrogen receptor positive) subgroup OR = 1.11, 95% CI = (1.03–1.20) and ER− (estrogen receptor negative) subgroup OR = 0.96, 95% CI = (0.84–1.10). These results indicated that, for the premenopausal group and ER+ group, the occurrence of breast cancer may be related to exposure to ELF-EMFs. However, for the postmenopausal group and ER− group, no relationship was observed. The results of this study were in accordance with the results of Girgert et al's studies. This will give us a big reason to make further study to assure whether ELF-EMFs affect the development of women's breast cancer by affecting hormones and what is the specific mechanism(s) behind this phenomenon.

In the epidemiologic studies, the result of correlation between ELF-EMF exposure and breast cancer in females was mainly affected by exposure assessment. There were only two studies taking living and working environment into consideration in the 23 studies. The rest studies only focused on one aspect of life or work such as usage of electric heating equipment (eg. electronic blanket), work duty and distance from the high voltage power lines. It was well known that the electrometric field was everywhere due to the wide-spread application of electric equipment. It was really hard to take all the exposures in living and working environment into consideration, however it was obvious that the exposure assessment was insufficient if only taking one aspect into account. The result of this meta-analysis suggested a significant difference (OR = 1.08, 95% CI = 1.00–1.15) for the occupational exposure subgroup was consistent with the result from T.C. Erren. Our study has especially conducted a meta-analysis on two studies with more comprehensive exposure assessment, OR = 1.35, 95% CI = 0.97–1.89,although statistical difference was not significant, the level of OR value indicated that more comprehensive studies need to be done on exposure assessment to discover the correlation between ELF-EMF exposure and breast cancer in females on top of the accuracy and reliability on case and control group selection. Our study has recommended the direction for future studies in this field.

From the results of this study, we determined that exposure to ELF-EMFs might be a risk factor contributing to the development of breast cancer, especially for premenopausal and ER+ females. Currently most of the studies have some defects in assessing and dividing exposure, therefore more studies with comprehensive and accurate exposure assessment are in need to further confirm the correlation between ELF-EMF exposure and breast cancer in females, Especially the combined effects of ELF-EMFs and estrogen, melatonin, or other hormones on the development of breast cancer.
